# The Influences of Drug Abuse on Mother-Infant Interaction Through the Lens of the Biopsychosocial Model of Health and Illness: A Review

**DOI:** 10.3389/fpubh.2019.00045

**Published:** 2019-03-12

**Authors:** Ilaria Cataldo, Atiqah Azhari, Aurora Coppola, Marc H. Bornstein, Gianluca Esposito

**Affiliations:** ^1^Affiliative Behavior and Physiology Lab, Department of Psychology and Cognitive Science, University of Trento, Rovereto, Italy; ^2^Mobile and Social Computing Lab, Bruno Kessler Foundation, Trento, Italy; ^3^Social and Affective Neuroscience Lab, School of Social Sciences, Nanyang Technological University, Singapore, Singapore; ^4^Psychology Unit, Azienda Provinciale per i Servizi Sanitari, Trento, Italy; ^5^Service for Addiction–Ser.D, Azienda Provinciale per i Servizi Sanitari, Trento, Italy; ^6^Child and Family Research, Eunice Kennedy Shriver National Institute of Child Health and Human Development, Bethesda, MD, United States; ^7^Institute for Fiscal Studies, London, United Kingdom

**Keywords:** substance use disorder, mothering, parenting, mothers, drug abuse, mother-infant, mother-infant interaction

## Abstract

Women who abuse illicit drugs often engage in atypical parenting behaviors that interfere with the natural development of mother-infant interaction and attachment. Maternal caregiving deficits leave pronounced adverse consequences in the wake of drug abuse relapse, which often occurs and in early infancy. These are times when the child requires optimal parental care. The contemporary literature documents long-term implications of illicit drug-abuse in parenting on infants. However, factors that drive and sustain the influence of drug abuse on parent-infant outcomes remain elusive. This review adopts a biopsychosocial approach to synthesizing the existing state of knowledge on this issue. Mother-infant interaction is a dynamic socio-relational process that occurs at multiple levels of organization. As such, a biopsychosocial perspective enables us to uncover: (i) roles of specific physiological mechanisms and biological characteristics of atypical parenting in mothers who abuse drugs, (ii) the influence of drugs on maternal psychological state (i.e., beliefs regarding parenting practices, emotional regulation), and (iii) social relationships (i.e., relationships with spouse and other drug abusers) and contextual cues (i.e., triggers) that moderate non-optimal maternal caregiving. A comprehensive review of these key domains provides a nuanced understanding of how these several sources interdependently shape atypical parent-infant interaction amongst drug abusing mothers. Systematic elucidation of major factors underlying drug-abused maternal behaviors facilitates the development of targeted and more effective interventions.

## 1. Introduction

Substance Use Disorder (SUD) is characterized by impairment in inhibitory control and social behaviors, risk taking, and hazardous pharmacological profiles, as defined in the fifth edition of the Diagnostic and Statistical Manual of Mental Disorder. SUD reflects the abuse of various illicit psychoactive drugs, such as cannabis, hallucinogens, opioids, stimulants (including cocaine), sedatives, and hypnotics (1). According to 2015 Statistics of the World Drug Report, a disturbing global trend of drug consumption has emerged showing that 247 million people abuse drugs; among them, 29 million have been diagnosed with drug use disorder, but only 1 in 6 actually started a rehabilitation programme (2). Data published on the National Institute on Drug Abuse (NIDA) (3) states that men are more likely to use illicit drugs compared to women, but women tend to present more severe clinical outcomes with regard to social, psychological, medical, and behavioral drug-related impairments (4). The difference in effects exerted by psychotropic substances between the sexes pivots on the differential influence of neuroactive steroid hormones for neurobehavioral outcomes (5). One notable sex distinction in the modulation of neural substrates is the potent influence of female hormones, estradiol and progesterone, on the striatal dopamine reward and attentional system (6). Hormonal involvement and modulation can partially explain dissimilarities between men and women in neural circuits of stress adaptation and reward, which drive drug-seeking behaviors (7).

When addressing issues of women and drug abuse, it is mandatory to consider pregnancy and parenting, and how they are affected by illicit substance consumption. Analyzing the different components of parenting in drug-abusing mothers from a biopsychosocial perspective can advance our understanding of the dynamics intervening between the individual and the context that drive behavioral change (8), and provide basis for understanding the determinants of disease and arriving at rational treatments and patterns of health care (9). The relation between the singular person and her multiple concurrent contexts is even more relevant in the postpartum period, which represents a critical phase for mothers. Numerous biological and environmental changes occur at this time, and this period represents the beginning of a temporal window during which parents and infants lay the foundation of attachment that endure and shape the individual's life-long socio-emotional competencies and stress regulatory capacities (10).

The aim of the current review is to summarize the state of the art about illicit drug-abuse on maternal practices and to uncover biological and physiological features of atypical mothering, the impact of illicit drug consumption on maternal psychological characteristics, and the influence of social relationships on the modulation of maternal behaviors.

### 1.1. Biopsychosocial Model of Drug Abuse and Parenting

SUD is characterized by a set of psychological and behavioral features which likely result from the development of tolerance, psychological and physiological dependence, and addiction. The persistence of addiction is due to mechanisms of reinforcement that can be both positive and negative. For example, positive reinforcement is the reward response that follows first consumption; a pleasurable experience with the drug leads to increased likelihood of further consumption. Conversely, the protracted use of the substance to avoid or soothe aversive withdrawal symptoms is considered a negative reinforcement that prolongs drug-intake behaviors and makes extinction more difficult (11). At a neurobiological level, reinforcement and relaps are modulated by both reward circuits and the stress response system (12). It is noteworthy that neurobiological changes occur in mothers' brains during the first few months after birth, mainly in brain regions designated to regulatory circuits, emotional responses, reward processing, executive functions, and parental behaviors (10). Processes implicated in drug addiction and mothering overlap at the neurobiological and psychological levels. Merging these considerations in the context of drug-abusing mothers renders it necessary to examine overall parent-infant interaction from a relational systems perspective that includes physiological and psychological needs of the mother, within a bioecological framework, so as to explicate the significance of autonomic mechanisms (13). Applying a physio-bioecological approach to the specific case of parenting in mothers with substance use disorders, the main aims are to uncover the effects that illicit drugs exert on maternal practices and parental styles in the domain of early mother-infant interaction, and to identify differences in maternal responses to infant stimuli between clinical and non-clinical populations.

## 2. Methods and Results

**Figure 1:**
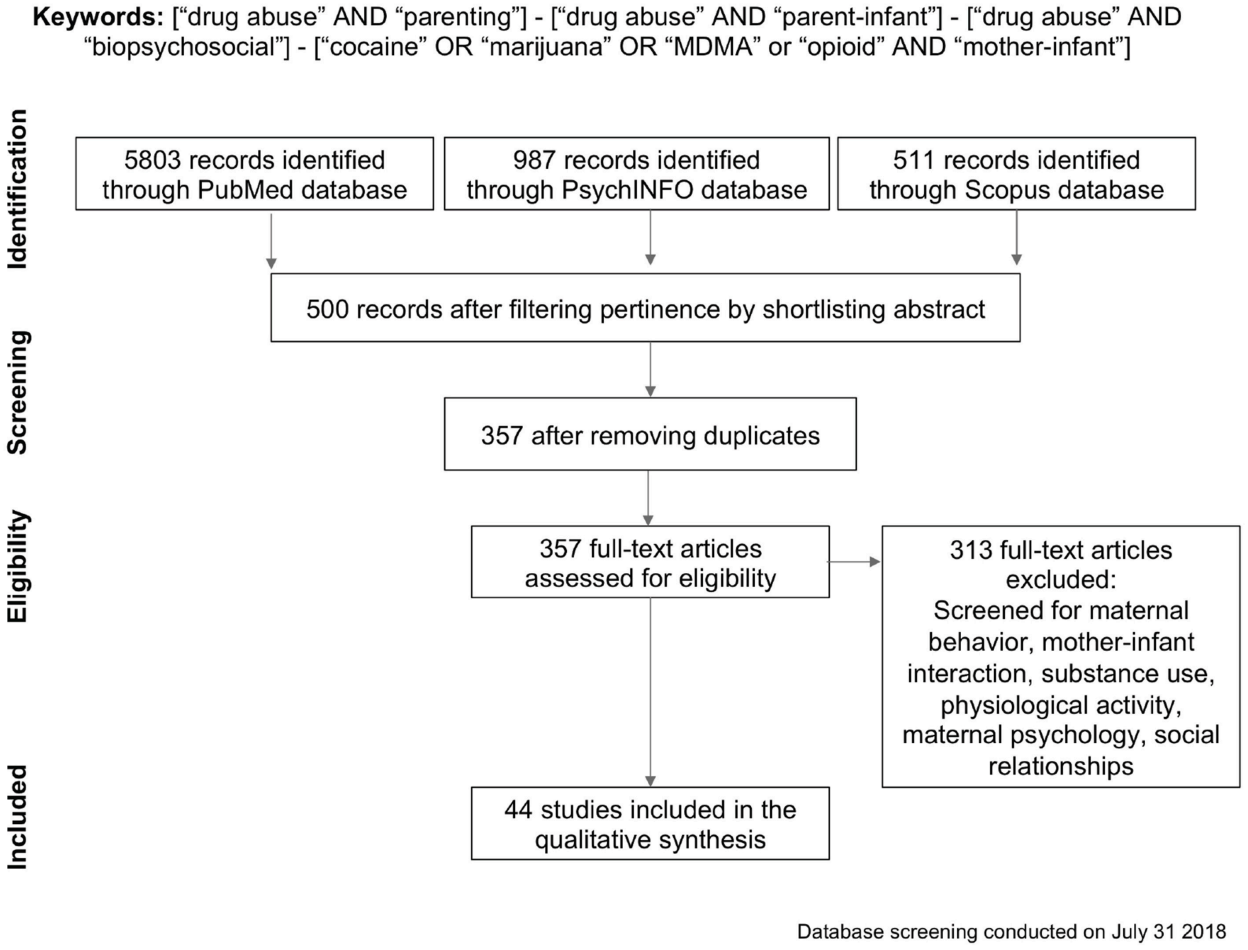
PRISMA flowchart for search criteria and papers eligibility.

We searched PubMed Central, PsycINFO and Scopus databases for articles on illicit drug abuse and parenting. We comparatively analyzed the entire literature from 1981 up to July 2018, combining different keywords and Boolean operators (see Figure 1 PRISMA flowchart). This database was generated by combining terms and Boolean operators, such as “drug abuse” AND “parenting,” “drug abuse” AND “parent-infant,” “drug abuse” AND “biopsychosocial.” To include more precise and targeted results, we conducted an additional search on the same databases using words describing the specific drugs we included in this review: “cocaine” OR “marijuana” OR “MDMA” or “opioid” AND “mother-infant.” Overall, 7301 papers were identified by merging the PubMed Central, PsychINFO and Scopus databases, including only peer-reviewed published journal articles. Articles were shortlisted according to their relevance, and duplicates were removed, resulting in 357 records which were subsequently checked for eligibility. Records about treatment programs, comorbidity with psychiatric disorders, fatherhood, ethical concerns, alcohol or nicotine, national policy, HIV, body mass index, adolescents or toddlers older than 3 years, work related issues, and service caregivers' perceptions of parenting practices were removed, as were articles which were not human studies, on drug abuse, or not focusing on mother-infant interaction. This screening restricted the database to 44 records for the qualitative analysis. Afterwards, these studies were labeled with the substance discussed and filtered depending on one of three major topics of interest: physiological and biological characteristics of atypical mothering, influence of drugs on maternal psychological state, and impact on mothers' social relationships (see Supplementary Table 1 for the list of papers included in the review).

### 2.1. Level 1-Physiological Mechanisms and Biological Characteristics of Atypical Parenting in Mothers Addicted to Drugs

Affiliative behaviors fall within the purview of the dopamine and oxytocin reward systems, which overlap with neural structures and pathways related to drug abuse and parental behavior. Hence, in mothers with issues pertaining to drug consumption, activation of one circuit may occur at the expense of the other. More concretely, the reward system could be involved in drug-seeking behaviors, with negative implications for mothering behaviors. Furthermore, brain areas related to perception and elaboration of infant cues, such as the prefrontal cortex, might be engaged in overcoming actions related to drug consumption, leading to subsequent higher levels of stress, thus affecting interactions with the infant (14).

In the case of cocaine-exposed mothers, neurohormonal pathways (especially those regulating oxytocin) can be altered, leading to decreased neurohormonal levels that affect neural responses to infant cues (15). Illicit substances impact motivation circuits implicated in parenting regulation (16) and maternal practices, such as infant feeding. In cocaine-abusing women, evidence points to a tendency for poor infant engagement (11) which deteriorates over the course of the first year of postnatal life (17); these effects are accompanied by shorter duration feeding sessions and diminished cognitive flexibility (18). Even among those receiving treatment, women exposed to opioids bear infants with neonatal abstinence syndrome and are less keen to breastfeed (19). Breastfeeding, while under opioid-treatment or not, has both short- and long-term consequences on dyadic attachment (20). SUD alters neurotransmission in the nucleus accumbens (NAcc), the prefrontal cortex (PFC), and ventral tegmental area (VTA). Functions in these areas become disrupted, with changes occurring in systems that regulate neurotransmitter levels in the forebrain and midbrain, like the transmission of serotonin and dopamine in the NAcc, and dysregulation of the hypothalamic-pituitary-adrenal axis (HPA) (12). These pathways enhance substance use relapse and augment negative affect, especially in women (4). Oxytocin modulates addiction-related behaviors, such as acquisition, withdrawal, drug-seeking, and relapse (15). Oxytocin contributes to social affiliative parenting behaviors. Not only is it involved in regulating uterine contractions during labor and milk ejection in breastfeeding, it is also pertinent to mother-infant bond formation and parental practices, eventually shaping the infant's own oxytocin profile (21). Although dopamine and oxytocin are different neurotransmitters, their pathways appear to be interlaced and, to a certain extent, overlap; thus, disruption of these systems can impact a multiplicity of mechanisms and behaviors related to both parenting and substance use (22). Indeed, during the early stages of development, infants express their needs through cries and facial expressions, and maternal drug consumption can alter maternal perceptions of these signals (23). For instance, modifications in perception of infant cues may manifest through a delay in facial recognition ERP responses to cry (24), and a reduction in activation of dopamine- and oxytocin-innervated brain regions while looking at their own baby's face (25, 26).

Drug addiction and motherhood are both accompanied by specific cerebral morphological and neurophysiological changes. Functional Magnetic Resonance Imaging (fMRI) studies on postpartum mothers' brains show an increase in gray matter volume in morphological structures, such as the hypothalamus, striatum, amygdala, thalamus, and insula, which are associated with the reward circuit, motivation, sensory information processing, emotional regulation, and empathy (26, 27). In an fMRI study, Landi and colleagues (14) compared neurophysiological activation patterns in response to infant stimuli between cocaine-exposed and non-exposed mothers. The clinical sample showed reduced neural activation in prefrontal areas, occipital lobes, and limbic structures (amygdala and parahippocampus) while looking at infant faces. The authors also reported decreased neural activation in the insula and auditory sensory areas while drug-abusing women were listening to infant cries (14). Prolonged diminished neural responses in these regions might compromise maternal behavior toward infants' needs and cues, with negative implications on mother-infant attachment (28).

### 2.2. Level 2-Influence of Drugs on Maternal Psychological State

Motherhood comes with a series of neurobiological modifications, representing a vulnerable temporal window for the development of depressive symptoms and changes in psychological states. We have reported studies highlighting the involvement of the reward circuit and amygdala in mechanisms motivating maternal behaviors (26, 27). These two areas are functionally connected to prefrontal cortical regions during emotion regulation through the use of cognitive strategies, such as reappraisal (29). The same substrates are implicated in drug consumption pathways that impair executive function, a multidimensional construct that includes, besides emotion regulation, a suite of cognitive operations like mental flexibility, inhibition, planning, working memory, reflective functioning (30), verbal fluency (31), and language (22, 32).

**Figure 2:**
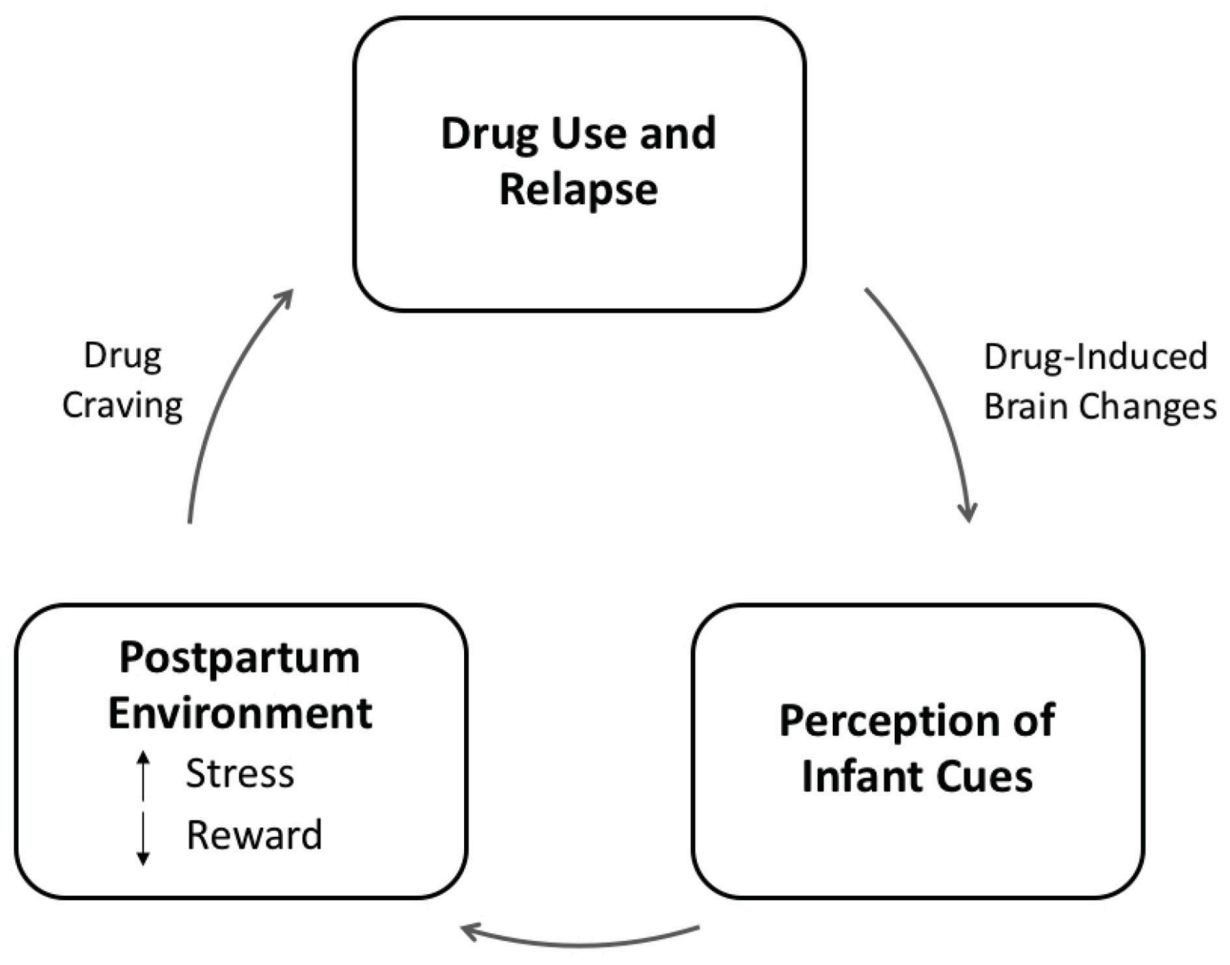
Model of the relation between addiction and parenting adapted from Rutherford et al. (11). In this model, addiction represents the dysregulation of stress and reward systems, both of which are adapted to support parenting. In the case of addiction, we propose that drug-induced brain changes result in the attenuation of the reward value of infant cues, which are replaced by a more stressful neurophysiological response. This stress response to infant cues may increase craving for drugs and promote drug seeking and relapse in abstinent mothers, thus perpetuating a cycle of neglect.

Struggles with emotion regulation in substance-abusing mothers might reinforce drug usage as a strategy to deal with stress, instead of developing or enhancing more adaptive regulatory skills, thus further aggravating emotional dysregulation (32). Difficulties in emotional regulation make it more challenging for drug-abusing mothers to maintain correct perceptions of their child's affective needs. At the same time, altered mechanisms of the reward circuit have implications for maternal parenting practices: for instance, the reward system might be more responsive to substance abuse, leading to maladaptive mother-infant interaction characterized by emotional disengagement and less responsive behaviors (24, 33, 34), (14, 35). Furthermore (16), quality of parenting might vary in a dose-dependent manner according to the quantity of drug consumed (17, 36). As a consequence, the demands for care which stand at the core of the mother-infant relationship may turn into a struggle, and fail to offer sufficient reward to addicted mothers, who are likely to adopt avoidant behaviors (11, 37) (see Figure 2).

Mother-infant attachment has become one of the most important concepts in developmental and clinical science since Bowlby published “Attachment and Loss” (38). Applying principles of attachment theory to the frame of substance-using mothers, individual characteristics of mother and infant alike define the nature of their interactions, so it is important to elucidate how specific drugs might alter mother-infant communication (39). Cocaine consumption during pregnancy compromises the quality of mother-infant interactions by altering maternal behaviors, such as warmth (40) and harshness (41), and infants of cocaine-abusing mothers are less responsive during play interactions (20, 24). Prenatally cocaine-exposed children undergo neurobehavioral changes, such as irritability, hypersensitivity, and difficulties in regulating emotional state that appear to prevent them from responding functionally to maternal stimuli; they may also appear lethargic, using sleepiness as a strategy to withdraw from stressful stimulation (42). Postnatal cocaine use has been reported to predict maternal insensitivity during interactions 8 weeks after birth (43). Overall, the quality of cocaine-mother and infant interaction is hallmarked by reduced mutual enjoyment, reciprocity, and regulation (33). However, some results in literature report that maternal cocaine use does not affect social interaction and attachment patterns at 12 (44) or 18 months (45). In general, an appropriate dyadic interaction requires high maternal sensitivity to infant cues. In this context, the concept of sensitivity implies the capacity to detect and accurately understand the child's signals and to respond appropriately according to the child's needs. This skill requires reflective abilities, such as mentalization, which appears to be impaired in drug-abusing mothers with regard to meeting the baby's needs and their own parental competencies (31). Frequently, mothers with low mentalizing capabilities attributable to substance use misunderstand infant behaviors, interpreting them as rejecting, and hence construct a representation of their child as intrusive, detached, or hostile (31, 42). Abuse of drugs during motherhood may result in “blue moods,” feelings of guilt, and inadequacy, which prevent the mother from being emotionally available in interactions with her offspring (42). Studies of drug-abusing mothers with more than one child have highlighted mothers' strain in delivering educational practices to older children, which point to difficulties in adjusting parenting behaviors that accord with developing requirements in their children (46).

### 2.3. Level 3-Social Relationships (i.e., Relationships With Spouse, Drug Abusers) and Contextual Cues (i.e., Triggers), in Modulating Maternal Misbehaviors

Drug abuse is related to the context of social relationships (especially when it develops into an addiction) due to its implications for users and for related people, such as families, other addicts, partners, and, of course, children (47). Substance-abusing mothers display more problematic behaviors during interactions and experience less social support and greater environmental difficulties, like domestic violence and other forms of abuse (48, 49). Socially rewarding experiences and relationships are protective factors against drug-seeking behaviors (50). Conversely, dysfunctional relationships can prompt drug abuse or relapse, especially during the perinatal and postpartum periods. During this time frame, there is a higher probability that women will become victims of intimate partner violence (IPV) (51), which is defined as the experience of “physical violence, sexual violence, stalking and psychological aggression, including coercive acts, by a current or former intimate partner” (52). When occurring during pregnancy, IPV has been correlated with adverse gestational events, such as preterm delivery and low weight at birth (53). Together with pregnancy intentions (e.g., unintended), IPV during the postpartum period is associated with increased use of substances in women as a mechanism to cope with stress (54, 55). Mothers who experience IPV often show hyper-controlling, overly permissive, or unresponsive maternal behaviors, along with poor emotional sustenance, leading to negative child developmental outcomes (51, 56). Mogro and colleagues reported that, contextually, a scarce social network can increase the risk of exposure and perpetration of IPV (57). Excepting some interventions, there is a notable gap in the literature about social support provided to drug-abusing mothers based on their social network; such support could represent a source of positive emotional help. Women with substance use issues have been reported to belong to limited social networks, providing them inadequate social support (58). Other contextual circumstances likely affect maternal behavior, such as the risk of losing custody of the child, which is twice as likely in substance-using compared to non-using mothers (59).

## 3. Prenatal Exposure to Drugs and Complications in Postnatal Period

Although not a main focus of the present review, it is important to note the effects of prenatal exposure to illicit drugs during pregnancy and in the the postnatal period. As fetal development proceeds very rapidly and being greatly influenced by intrauterine environment and maternal behavior, maternal SUD may disrupt formation of several systems. Drugs might interrupt normal presynaptic reuptake of neurotransmitters (i.e., dopamine, serotonin), causing greater concentrations in the extracellular environment and risk of abnormal brain development (60).

Such consequences are generally associated with a set of medical conditions, including physical development, such as alterations of normal fetal growth, length and weight (61, 62) and morphometric cerebral features (63), but specific outcomes on perinatal and postpartum phases differ according to the substance the fetus has been exposed to. With regards to methamphetamine exposure, newborns might show congenital abnormalities like cardiac alterations and withdrawal symptoms (64). Maternal cocaine use during pregnancy might lead to intrauterine growth retardation and medical outcomes at birth, such as seizures, vomit, and alterations in sleep and cry patterns (62). In opioid-dependent women, who are usually subjected to methadone-maintenance therapy during gestation, neonatal issues appear to be quite severe, with a very high percentage of infants born prematurely and experiencing neonatal abstinence syndrome (NAS) in the first two weeks (65).

These complications have a profound impact on the prenatal and perinatal periods and have consequences in the long term. As mentioned, *in utero* drug exposure affects fetal development also due to the alteration of molecular pathway and neurobiology, such as cortical thickness, however long-term neurobehavioral concerns have been observed in children of drug-abusing mothers, including deficits in cognitive performance and conduct related issues, like negative reactivity and altered arousal and emotional problems (63, 66). Despite great progress in research in the last decades, some results appear inconsistent because of different factors (i.e., type of substance, quantity and frequency of intake) and possible co-presence of confounding factors (64, 67). Thanks to the new methodological approaches, together with longitudinal studies and animal models, it will be possible to broaden and deepen the understanding of dose-related issues and develop specific protocols of biopsychosocial interventions to attenuate the impact of prenatal drug exposure on future risk.

## 4. Maternal Drug Abuse and Risk for Child Maltreatment

Combining biological and psychological factors occurring in maternal substance use disorder discussed so far (of both mother and infant) with a challenging environment, it is possible to have a wider perspective of the complex frame, wherein the mother-infant dyad generates its bond. Issues related to parenting abilities in drug-abusing women are a great concern under a psychosocial perspective, not only with regards to women's mental health, but also to child development. In the model proposed in this review, emotional regulation in drug-abusing mothers is impaired due to substance consumption, which might lead to craving and drug-seeking behaviors to ease stress derived from infant cues, thereby enhancing maladaptive parental practices, sometimes at the expense of the well-being and safety of the child (48). Much research supports the association between prenatal drug exposure and childhood outcomes, but still few studies focus on maternal substance and child maltreatment, highlighting the increased risk of abuse (68), especially when combined with parental depressive symptoms (69, 70). As there is no standardized protocol for data collection in this specific field, statistics account for estimates that mainly rely on self-report information coming from intervention programs for drug-abusing mothers, describing a sample that hardly represent the actual one and that are more oriented to child protection than rehabilitation from SUD; more data derive from child welfare services, that usually adopt observational protocols focused on parenting abilities [for a review, see (71)]. The percentage of parents with only substance-use related issues involved in child welfare services is relatively small (72). This highlights the needs for a deeper comprehension of each component and more intensive focus on biophysiological influences and consequences to provide more tailored interventions within the dyad.

## 5. Discussion and Conclusion

In this review, we focused on how parenting, which is already stressful, affects and is affected by Substance Use Disorder, which has a large incidence in the general population. When a fundamental human relationship, like mother-infant interaction, intersects with a complex construct, like substance abuse, it is essential to consider all facets of the issue within a multilevel approach, such as what we have employed here.

Although parenting and drug-abuse behaviors operate on common brain regions and neurohormonal circuits (11, 14), the manner in which they impact life can vary across women. Both parental and drug-abusing behaviors are dynamic in nature, shaped by interactions with external cues (infant needs or craving for substance) and changeable patterns of action. To better understand the behavioral outcomes of the overlap of motherhood and drug-related issues, it is crucial to analyze the elements of which they are composed within a biopsychosocial framework separately, so as to define effective features of these occurrences. In attempting to extend this knowledge, we have uncovered several gaps in the literature.

First, substance use is strictly connected with laws and norms, especially when considering the consumption of illicit drugs, such as cocaine, heroin, hallucinogens, and methamphetamine. This law-related factor highlights a critical issue of research in illicit drug use, misuse, and abuse, revealing an important gap. In fact, data are mainly collected using self-report questionnaires or provided by mental and social services. This method of data collection often yields an unrealistic estimate of the problem, which likley appears underreported.

Second, studies in the existing literature present data from USA, South America, Africa, and Europe, leaving the issue poorly explored in Asian and Pacific countries. Although a few reports display some prevalence rates, these are mainly estimates (73).

Illicit drug use acts on specific brain structures, where each substance exerts distinctive effects, altering perceptions in ways that could compromise maternal parenting practices. Only a few studies have compared differences in mother-infant interactions among diverse drug choices (24, 46); they tend to show variation in maternal engagement and responsiveness during interactions. Only one study distinguished abusers on the basis of drug quantity consumption (17), highlighting a more severe impairment in heavy consumers. Outcomes in both maternal and infant engagement and responsiveness while interacting may also vary depending on the age of the mother and years of drug consumption prior to pregnancy.

As emotion and stress regulation are some main mechanisms involved in parenting, it is desirable to provide more evidence about physio-behavioral responses to infant stimuli, such as promptness to action or measures of hormonal levels, in drug-using and non-using mothers and across different substances, to further elucidate their respective effects on parenting. Our search resulted in only a few studies that assessed physiological responses, such as electrical brain activity (24), whereas most focused on functional brain activation patterns (14, 26).

Most research in this field aims to reduce early life adversities, intergenerational effects, and the perpetuation of the cycle of addiction and childhood neglect (27), but there is a great need for more evidence related to physiological consequences of substance abuse on mothering (e.g., promptness to action, stress perception) to overcome self-report information and achieve a more reliable picture of developmental outcomes. The availability of more reliable information would lead to the possibility of more customized clinical practice and intervention with pregnant drug abusing women and for the mother-child dyad.

## Author Contributions

IC, AA, AC, MB, and GE conceived and designed the paper. IC and AA reviewed the literature and wrote the paper. MB and GE commented and submitted the paper.

### Conflict of Interest Statement

The authors declare that the research was conducted in the absence of any commercial or financial relationships that could be construed as a potential conflict of interest.
