# Supplementary material for: The Influences of Drug Abuse on Mother-Infant Interaction Through the Lens of the Biopsychosocial Model of Health and Illness: A Review
Source: Front Public Health. 2019 Mar 12;7:45. doi: 10.3389/fpubh.2019.00045 (PMC6422866; doi:10.3389/fpubh.2019.00045)
Supplement: Supplementary file 1 [file Data_Sheet_1.pdf]

Supplementary Material:

Through the lens of the biopsychosocial model of health and illness: A review on the influence of drug abuse on Mother-Infant interaction

1 SUPPLEMENTARY TABLE 1

Table S1.

	Author/s	Year	Topic	Substance/s	Child's Age
1	Brancato and Cannizzaro	2018	Hormonal correlates of maternal behaviour	multiple	perinatal
2	Suchman et al.	2017	Mother mentalization	multiple	1-5 years
3	Kim S. et al.	2017	Early adverstities	not specified	perinatal / infancy
4	Kim S. et al.	2017	Maternal brain acivation	cocaine	6 months
5	Rasmussen et al.	2016	Emotional disengagement	multiple	2-44 months
6	Kim P. et al.	2016	Brain plasticity	not specified	birth - infancy
7	Parolin and Simonelli	2016	Adult and infant attachment	not specified	pregnancy - infancy
8	Eidenet al.	2015	Child self-regulation and maternal harshness	cocaine	0-4 years
9	HŒkansson et al.	2015	Mother mentalization and executive functioning	not specified	6-18 months
10	Piallini et al.	2015	Brain activation	not specified	infancy
11	Moses-Kolko et al.	2014	Postpartum psychopathology effect on caregiving	not specified	perinatal
12	Eiden et al.	2014	Internalizing behavior problems	cocaine	0-12 years
13	Schuetze et al.	2014	Responsivity to the child	cocaine	0-23 months
14	Eiden et al.	2014	Child self-regulation and maternal harshness	cocaine	0-23 months
15	Williams and Johns	2014	Hormnal correlates and maternal behavior	cocaine	infancy
16	Alhusen et al.	2013	Intimate partner violence	marijuana	pregnancy - infancy
17	Borelli et al.	2012	Maternal avoidant language	methadone	0-36 months
18	Pajulo M. et al.	2012	Reflective functioning	not specified	pregnancy - infancy
19	Haltigan et al.	2012	Attachment security	cocaine	18 months
20	Landi et al.	2011	Brain responses to infant cries	multiple	infancy

	Author/s	Year	Topic	Substance/s	Child's Age
21	Eiden et al.	2011	Maternal sensitivity and affect	cocaine	pregnancy - 13 months
22	Rutherford et al.	2011	Brain and hormonal correlates	cocaine	postnatal
23	Eiden et al.	2011	Maternal warmth and child behavior problem	cocaine	1-18 months
24	Eiden et al.	2011	Child self-regulation and maternal harshness	cocaine	3 years
25	Eiden et al.	2011	Child behavioral problems and maternal warmth	cocaine	0-18 months
26	Eiden et al.	2011	Maternal psychological distress	cocaine	13 months
27	Greenfield et al.	2010	Maternal biological issues	multiple	pregnancy - infancy
28	Strathearn and Mayes	2010	Hormnal correlates and maternal behavior	cocaine	pregnancy - infancy
29	Swain et al.	2007	Brain activation	cocaine	postnatal
30	Eiden et al.	2006	Maternal sensitivity and affect	cocaine	4-8 weeks
31	Tronick et al.	2005	Maternal behavior engagement	multiple	0-23 months
32	Minnes et al.	2005	Quality of interaction	cocaine	0-12 months
33	Quinlivan and Evans	2005	Domestic Violence	cocaine	6 months
34	Swain et al.	2005	Early Attachment	opiates	infancy
35	Schuler et al.	2002	Parenting attitudes	multiple	0-18 months
36	Pajulo M. et al.	2001	Maternal perception of social support	not specified	3-6 months
37	Eiden et al.	2001	Feeding interactions	cocaine	2 months
38	Ukeje et al.	2001	Attachment security	cocaine	12 months
39	Blackwell et al.	1998	Quality of interaction	cocaine	4-6 months
40	Mayes et al.	1997	Maternal attentiveness and responsiveness	cocaine	3-6 months
41	Miller	1997	Maternal variables	cocaine	infancy
42	Heller et al.	1996	Quality of verbal/non-verbal behaviors	cocaine	3-4.5 years
43	Gottwald and Thurman	1994	Maternal engagement and infant attentiveness	cocaine	1 day
44	Black et al.	1993	Neurodevelopmental patterns	cocaine	0-6 weeks
45	Mayes et al.	1992	Neurodevelopmental patterns	cocaine	pregnancy - infancy
46	Burns K. et al.	1991	Quality of interaction	cocaine	8-11 months
